# Parthenolide ameliorates 3-nitropropionic acid-induced Huntington’s disease-like aberrations via modulating NLRP3 inflammasome, reducing microglial activation and inducing astrocyte shifting

**DOI:** 10.1186/s10020-024-00917-5

**Published:** 2024-09-26

**Authors:** Mona E. Noureldeen, Nancy N. Shahin, Hebat Allah A. Amin, Maha M. El-Sawalhi, Heba R. Ghaiad

**Affiliations:** 1 Biochemistry Department, Faculty of Pharmacy, Cairo University, Kasr El-Ainy St., Cairo, 11562 Egypt; 2 Pathology Department, Faculty of Medicine, Helwan University, Cairo, 11795 Egypt

**Keywords:** Huntington’s disease, 3-Nitropropionic acid, Neuroinflammation, NLRP3, NF-κB, Parthenolide

## Abstract

**Background:**

Huntington’s disease (HD) is a progressive neurodegenerative disease that causes motor, cognitive, and psychiatric abnormalities, with no satisfying disease-modifying therapy so far. 3-nitropropionic acid (3NP) induces behavioural deficits, together with biochemical and histological alterations in animals’ striata that mimic HD. The role of nucleotide-binding domain, leucine-rich–containing family, pyrin domain–containing-3 (NLRP3) inflammasome in HD pathogenesis remains largely uncharacterized. Parthenolide (PTL), a naturally occurring nuclear factor kappa B (NF-κB) inhibitor, is also known to inhibit NLRP3 inflammasome. Whether PTL is beneficial in HD has not been established yet.

**Aim:**

This study evaluated the possible neuroprotective effects of PTL against 3NP-induced behavioural abnormalities, striatal biochemical derangements, and histological aberrations.

**Methods:**

Male Wistar rats received PTL (0.5 mg/kg/day, i.p) for 3 weeks and 3NP (10 mg/kg/day, i.p) was administered alongside for the latter 2 weeks to induce HD. Finally, animals were subjected to open-field, Morris water maze and rotarod tests. Rat striata were examined histologically, striatal protein expression levels of glial fibrillary acidic protein (GFAP), cluster of differentiation 45 (CD45) and neuron-specific enolase (NSE) were evaluated immunohistochemically, while those of interleukin (IL)-1β, IL-18, ionized calcium-binding adapter molecule-1 (Iba1) and glutamate were determined by ELISA. Striatal nuclear factor erythroid 2-related factor 2 (Nrf2), Kelch-like ECH-associated protein (Keap1), NF-κB, NLRP3, apoptosis-associated speck-like protein containing a CARD (ASC), caspase-1, S100 calcium-binding protein A10 (S100A10) and complement-3 (C3) were assessed by gene expression analysis.

**Results:**

PTL improved motor, locomotor, cognitive and anxiety-like behaviours, restored neuronal integrity, upregulated Nrf2, and inhibited NLRP3 inflammasome, NF-κB and microglial activation. Additionally, PTL induced astrocyte shifting towards the neuroprotective A2 phenotype.

**Conclusion:**

PTL exhibits neuroprotection against 3NP-induced HD, that might be ascribed, at least in part, to its modulatory effects on Keap1/Nrf2 and NF-κB/NLRP3 inflammasome signaling.

**Supplementary Information:**

The online version contains supplementary material available at 10.1186/s10020-024-00917-5.

## Introduction

Huntington’s disease (HD) is a devastating dominant autosomal neurodegenerative disorder with a prevalence of 5 cases in 100,000 people (Illarioshkin et al. 2018; Medina et al. 2022). HD is distinguished by a triad of motor abnormalities, cognitive derangements, and psychiatric disturbances that impede HD patients from carrying out their regular daily chores. A stretched CAG nucleotide repeat in the huntingtin (HTT) gene, that encodes an abnormal polyglutamine expansion in the HTT protein, is known to be the cause of HD (Baig et al. 2016; Eje et al. 2023). Such polyglutamine overexpression is responsible for the formation of insoluble aggregates which is the principal contributor in the manifestation of HD. These insoluble aggregates are also responsible for secondary ramifications, including mitochondrial dysfunction, glutamate excitotoxicity and neuroinflammation (Illarioshkin et al. 2018; Kumar et al. 2020). One major feature of HD is striatal neuronal death especially as the striatum is involved in motor control as well as learning functions (Wiprich and Bonan 2021).

Normally, there are several mechanisms through which astrocytes support neurons, such as neurotrophic support, glutamate uptake, and potassium buffering. On the other hand, reactive astrogliosis associated with neurodegenerative disorders is responsible for tampering astrocyte activities whereby astrocytes do not support neurons and release detrimental factors with a simultaneous increase in glial fibrillary acidic protein (GFAP) (Liddelow et al. 2017; Simpson et al. 2010).

Another hallmark in HD pathogenesis is the astrocyte shift from the neuroprotective phenotype, A2, into the neurotoxic A1 phenotype. Several indicators were reported to be A2 astrocyte-specific, including the S100 calcium-binding protein A10 (S100A10) and pentraxin-3 (PTX3), whereas others were known to be A1 astrocyte-specific, most notably the complement component 3 (C3) and guanine nucleotide binding protein 2 (GBP2) (Fan and Huo 2021; King et al. 2020; Liddelow et al. 2017).

A1 astrocytes express many neurotoxic genes responsible for microglial activation (Liddelow et al. 2017). Indeed, microglial activation has been found in HD brain and was considered as a powerful pathogenic element of neuroinflammation in HD (Crotti and Glass 2015). Activation of microglia is reportedly evoked by the mutant huntingtin protein (mHTT)-mediated excitotoxicity in microglia of HD patients (Yang et al. 2017). Ionized calcium binding adapter molecule-1 (Iba1) protein catalyzes the bundling of actin filaments and membrane ruffling with subsequent phagocytosis in activated microglia, while cluster of differentiation 45 (CD45) is known to occur on the microglial surface and to be upregulated upon microglial activation (Huang et al. 2016). Both Iba1 and CD45 are used as markers of microglia and active microgliosis (Honarpisheh et al. 2020; Korzhevskii and Kirik 2016) .

Experimental models using rodents have been extensively adopted for investigating the pathogenesis of HD and the outcomes of potential therapeutic strategies. Non-genetic animal models are known to incite cellular death either by dysregulating mitochondrial machinery or through excitotoxicity. The naturally occurring mycotoxin 3-nitropropionic acid (3NP) produced by plants is used to induce HD-like symptoms in rats (El-Sahar et al. 2020; Ramaswamy et al. 2007). 3NP is an irreversible inhibitor of succinate dehydrogenase (SDH) that instigates mitochondrial impairment and sparks reactive oxygen species (ROS) overproduction together with a decrease in the cellular antioxidant defenses. The systemic administration of 3NP is known to instigate a mitochondrial dysfunction accompanied with neuronal damage to the striatum, basal ganglia, hippocampus, spinal tracts and peripheral nerves in experimental animals (Bansal and Deshmukh 2018; Ludolph et al. 1991). Moreover, 3NP creates an inflammatory milieu by enhancing inflammatory cytokine release (Jang et al. 2013) and activating nuclear factor kappa B (NF-κB) signaling (Mansour et al. 2022). Furthermore, 3NP administration has been associated with downregulation of nuclear factor erythroid 2-related factor 2 (Nrf2), the master orchestrator of cellular defense against oxidants, coupled with upregulation of Kelch-like ECH-associated protein (Keap1), responsible for suppression of Nrf2, leading to augmented oxidative stress, neuroinflammation, and apoptosis (Jang and Cho 2016; Ma 2013). Moreover, 3NP intoxication provokes elevations in the excitatory neurotransmitter glutamate (Abdelfattah et al. 2020) and neuron-specific enolase (NSE), an efficient indicator of neuronal damage and neuronal cell death (Virdi et al. 2021).

Activation of the NLRP3 inflammasome can be triggered by aggregated and misfolded proteins, like mHTT, and mitochondrial impairment (Shao et al. 2018). Inflammasomes are multiprotein complexes located in the cytoplasm which, upon assembly, trigger the activation of caspase-1 that is accountable for the maturation and release of the inflammatory cytokines, interleukin (IL)-1β and IL-18, finally eliciting pyroptosis, a lytic cell death mode that releases other inflammatory mediators (Ali et al. 2024; Voet et al. 2019). NLRP3 inflammasome activation seems to involve two stages. The first is the NF-κB-mediated signaling activation, which consequently triggers the transcription of inactive NLRP3, proIL-1β and proIL-18. The second stage involves the formation of a cytosolic complex consisting of oligomerized NLRP3 assembled with ASC and procaspase-1. This assembly induces the conversion of procaspase-1 into caspase-1, and the subsequent release of mature IL-1β and IL-18 (Shao et al. 2018). Various molecular mechanisms have been postulated to demonstrate how the activation of NLRP3 can drive the activation of caspase-1 and the maturation of IL-1β and IL-18. These mechanisms include pore formation and potassium ion efflux, destabilization and rupture of lysosomes, and finally mitochondrial ROS production. Moreover, several studies have demonstrated that in NLRP3 inflammasome-associated autoinflammatory diseases, cell stress promoted the release of ATP and sustained elevated blood concentrations of IL-1β and IL-18 (Carta et al. 2015; Jo et al. 2016). Activation of caspase-1 has been detected in both HD patients’ brains and animal models of HD (Chen et al. 2022; Ona et al. 1999; Voet et al. 2019). Therefore, the NLRP3 inflammasome could be a druggable target to be used in the treatment of HD (Voet et al. 2019).

Parthenolide (PTL) is a naturally occurring sesquiterpene lactone that is one of the main compounds in feverfew, also known as *Tanacetum parthenium*, which has been traditionally used as a medicinal herb to treat a variety of diseases (Pareek et al. 2011). PTL has potent antioxidant and anti-inflammatory activities and can pass the blood brain barrier (BBB) (Ghantous et al. 2013). The anti-inflammatory effects of PTL are linked to its ability to inhibit NF-κB signaling (Gaojian et al. 2020; Khare et al. 2017). Moreover, PTL is a well-established inhibitor of NLRP3 inflammasome and its inflammatory cascade (Ding et al. 2022; Juliana et al. 2010; Zahid et al. 2019), via alkylation of the cysteine residues in the ATPase domain of NLRP3 and in caspase-1 (Coll et al. 2015). PTL has been demonstrated to improve motor, cognitive and behavioural dysfunction, inhibit neuroinflammation, and show potent neuroprotective effects in traumatic brain injury (TBI) (Ding et al. 2022) and Alzheimer’s disease (AD) (Fan et al. 2023; Sun et al. 2023), among many other neurological disorders (Cui et al. 2021; Dong et al. 2013; Fan et al. 2023). So, this study was directed to assess the neuroprotective role of the NLRP3 inflammasome inhibitor, PTL, against 3NP-induced behavioural abnormalities, striatal biochemical derangements, and histological changes.

## Materials and methods

### Animals

This study was carried out on a total of 64 adult male Wistar rats, with an average weight of 180 ± 20 g, that were obtained from the animal house of the National Cancer Institute, Cairo, Egypt. The rats were given two weeks to adapt before starting the experiment and were housed in controlled temperature and humidity conditions with 12 h light/dark cycle for the whole experimental period. Food and water were freely allowed throughout the study. All animal handling and experimentation protocols were conducted according to the Guide for Care and Use of Laboratory Animals issued by the US National Institute of Health (NIH Publication No. 85 − 23, revised 1996) and were consented by the Research Ethics Committee for Animal Experimentation at Faculty of Pharmacy, Cairo University (REC-FOPCU), with permit number: BC [3028].

### Chemicals

3NP was acquired from Sigma Aldrich Chemical Co. (cat. no. N22908, USA). PTL was obtained from Santa Cruz Biotechnology (cat. no. sc-3523, USA). Other chemicals used were of the highest analytical grade and were provided by Sigma-Aldrich (USA) or Merck (Germany).

### Experimental design

**Fig. 1:**
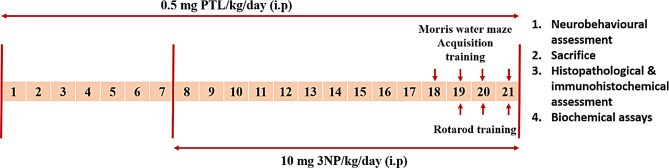
Timeline schedule for the experimental study. 3NP: 3-nitropropionic acid; PTL: parthenolide; i.p: intraperitoneal

Rats were randomly classified into 4 groups (16 rats per group) as summarized in Fig. 1. Group 1 (Normal Control) received daily intraperitoneal (i.p.) injections of 1% dimethyl sulfoxide (DMSO) for 21 days, in addition to daily i.p. injections of phosphate buffered saline (PBS), for 14 days starting from day 8. DMSO was administered one hour before saline administration. Group 2 (PTL) served as the drug control group, and received i.p. injections of 0.5 mg/kg/day PTL, dissolved in DMSO, for 21 days (Bahabadi et al. 2017), and starting from day 8, an additional i.p. injection of PBS was also administered for 14 days. PTL was given one hour before PBS administration. Group 3 (3NP-intoxicated group) received daily i.p. injections of DMSO for 21 days, and starting from day 8, the animals received i.p. injections of 10 mg/kg 3NP, dissolved in PBS, daily for 14 days (Túnez et al. 2010). Group 4 (PTL + 3NP) received 0.5 mg/kg/day PTL i.p. for 21 days, and starting from day 8, the rats received 3NP one hour after PTL injection.

### Sampling

After 21 days, all experimental animals were subjected to neurobehavioural assessment. Then, they were euthanized by cervical dislocation under anaesthesia, using 40 mg/kg phenobarbitone (i.p.) (El-Sahar et al. 2020), followed by decapitation. Subsequently, brains were exposed. Accordingly, each group’s rats were subsequently allocated into 2 subsets. In the first subset (*n* = 4), brains were immediately fixed in 10% neutral-buffered formalin for 24 h for the histopathological and immunohistochemical examination. In the second subset (*n* = 12), the striata were rapidly dissected on ice then washed twice with ice-cold PBS; one striatum from each rat was used to assess biochemical endpoints by ELISA technique, while the other striatum was used for gene expression analysis by quantitative reverse transcription polymerase chain reaction (qRT-PCR).

### Neurobehavioural assessment

At the end of the experimental period, the open-field test was used to evaluate motor function and anxiety-like behaviour, the rotarod test was employed as a measure of locomotor coordination (Carter et al. 2001; Knardahl and Sagvolden 1979), and finally Morris water maze was applied to evaluate spatial learning and memory (Morris 1984). The neurobehavioural tests were carried out in the same above-mentioned order, from the least to the most stressful, with a 2-hour resting interval between the tests. The movement of rats in the open-field and Morris water maze was recorded by means of ANY-Maze video tracking software (Stoelting Co, USA).

### The open-field test

The open-field test was carried out to assess spontaneous motor functions and anxiety-like behaviours in rats (Knardahl and Sagvolden 1979). The test was conducted in a sound-insulated room in which each rat was gently placed in the centre of a box where it was allowed to explore for 180 s. During the assigned time, the rat’s behaviour was monitored by a fixed overhead camera to determine the following measurements: mean speed, total distance travelled, number of squares crossed, immobility time, in addition to the number of times the animal is sitting up with both of its front paws and head raised and sniffing, known as rearing frequency (Gould et al. 2009). Additionally, the distance travelled and the time the rats were active in the central squares were determined. Finally, the time spent in close proximity to the walls, a phenomenon referred to as thigmotaxis time, was also recorded (Ávila et al. 2010; Gould et al. 2009).

### The rotarod test

Rat motor coordination and balance were evaluated using the rotarod test (Carter et al. 2001). Prior to the test day, a 3-days training regimen was conducted for each rat during which the animal was put back onto the rotarod every time it slid off (Jones and Roberts 1968). The latency time for the rat to fall off the rotarod was recorded and the cutoff time was 300 s. The fall-off time was recorded on the test day following the open-field test (Sekar et al. 2020).

### Morris water maze

The Morris water maze paradigm was adopted as a measure of spatial learning and memory retention (D’Hooge and De Deyn 2001). Three training sessions were conducted every day on rats, 120 s each, for 4 successive days. Throughout each acquisition trial, rats were allowed to reach the platform in the target quadrant freely. Once the rat found the platform, it was allowed to stay on it for 10 s. Upon failure to locate the target during a period of 120 s, the rat was gently put on the platform for another 30 s (Morris 1984). A probe test was carried out on day 5, wherein the platform was taken away, each animal was placed in the water facing the pool wall beginning in the quadrant opposite to the platform quadrant and was free to go around the pool for 60 s. Using an overhead camera, the durations of swimming in the target quadrant and the platform zone were noted (Kumar et al. 2011).

### Enzyme-Linked Immunosorbent Assay (ELISA)

Striata were homogenized in ice-cold PBS to yield 10% aqueous homogenate using T10 basic ULTRA – TURRAX (IKA^®^, Germany). Striatal levels of IL-1β, IL-18 and Iba1 were assessed by rat IL-1β ELISA kit (cat. no. E-EL-R0012), rat IL-18 ELISA kit (cat. no. E-EL-R0567) and rat allograft inflammatory factor ELISA kit (cat. no. E-EL-R1110), respectively, obtained from Elabscience Biotechnology (China). IL-1β, IL-18 and Iba1 concentrations were expressed as nanomoles per mg protein. Glutamate was quantified in striatal tissues using rat glutamate ELISA kit (cat. no. EK721805) obtained from AFG BioScience (USA). The concentrations of glutamate were expressed as nanomoles per mg protein.

### Protein estimation

The protein content of striatal tissue homogenates used for ELISA was assayed using a bicinchoninic acid (BCA) protein assay kit (cat. no. BCA1, Sigma-Aldrich, USA) as specified by the manufacturer and according to the method of Smith et al. (Smith et al. 1985).

### Quantitative reverse transcription polymerase chain reaction (qRT-PCR)

Striata were homogenized in a lysis buffer supplemented with β-mercaptoethanol using T10 basic ULTRA – TURRAX, (IKA^®^, Germany). Total RNA was extracted from striatal tissue using GeneJET RNA purification kit (cat. no. K0731, Thermo Fischer Scientific, USA) complying with the manufacturer’s guidance. The concentration and purity of the isolated RNA were measured according to the 260/280 nm absorption ratio by spectrophotometry. Afterwards, reverse transcription was achieved using a RevertAid first strand cDNA synthesis kit (cat. no. K1622, Thermo Fisher Scientific, USA) following the manufacturer’s recommendations. The quantitative real-time polymerase chain reaction was conducted via Maxima SYBR Green qPCR Master Mix kit (cat. no. K0251, Thermo Fisher Scientific, USA) in accordance with the manufacturer’s procedure. Primer design was accomplished via NCBI primer Blast and custom-made by Invitrogen (USA). The used primer sequences are listed in Table 1. The fold change in the expression of the target genes was computed based on the 2^−ΔΔCT^ formula with β-actin as a reference gene (Livak and Schmittgen 2001).

**Table 1:** The sequences of primers used for real-time PCR analysis

Gene name	NCBI Accession Number	Primer sequence
NLRP3	NM_001191642.1	Forward 5’CAA ACT AAG GCC CCG TCC AT 3’ Reverse 5’ACG TGC CTA GAA GGA AAG CC 3’
ASC	NM_172322.2	Forward 5’GTG GGA GTC AAC CCC GAA AG 3’ Reverse 5’AGC AGA GAC ACT GGT TGC AG 3’
S100A10	NM_031114.1	Forward 5’CTA CTT GAC AAA GGA GGA CCT GA 3’ Reverse 5’CAC AGC TTC GCA AGT AGT GAC C 3’
Complement Component 3	NM_016994.2	Forward 5’TAG ATG GGT GGT CGT GCA TC 3’ Reverse 5’GCT CCA GGT CTC GCT TCT T 3’
Caspase-1	NM_012762.3	Forward 5’GGG CTC GTT TGG GGA ATA AG 3’ Reverse 5’GCC AGA CAC TAC GGC TTC A 3’
Nrf2	NM_001399173.1	Forward 5’GTT CGG GTG GAC GTG GAT AC 3’ Reverse 5’AGG GTG GAT ACC ATA GCC GT 3’
Keap1	NM_057152.2	Forward 5’GAG TCC GAG GTG TTC CAT GC 3’ Reverse 5’CAC CAG GTA GTC CTT GCA GC 3’
NF-κB p65	NM_001415012.1	Forward 5’GCC CAC GTT CCA TGC TTT AC 3’ Reverse 5’TCC CTA ACT ACC AGC GGT CT 3’

## Histopathological examination

### Hematoxylin and eosin (H&E) evaluation and neuronal count determination

Four rats were arbitrarily chosen from each experimental group for histological evaluation of their brains. The whole brains were dissected and pre-fixed in 10% neutral-buffered formalin for 24 h. The brain was coronally sectioned at the level of the striatum. Two − 0.4 cm slices, rostral and caudal to the midline, were sliced from each brain and examined. The samples were dehydrated by passing through serial dilutions of alcohol, cleared in xylene and embedded in paraffin. Prepared paraffin blocks were cut 3 μm thick by a Leica RM 2155 microtome (USA). The obtained tissue sections were collected and mounted on glass slides, stained by hematoxylin and eosin (H&E) to microscopically examine the striatal regions by a light microscope (Woodruff et al. 2006).

Different cells were identified morphologically, following the morphological criteria for distinguishing neurons from glial cells in the nervous tissue previously described by Kołodziejczyk et al. (2014) and García-Cabezas et al. (2016). Neurons are known to have euchromatin in the nucleus, a clearly visible nucleolus with surrounding cytoplasm with a large cell body, while glia are known to be smaller with heterochromatin in the nucleus and no cytoplasm, thus a small rim of cytoplasm circling the entire nucleus is a useful feature to distinguish small neurons from astrocytes (García-Cabezas et al. 2016; Kołodziejczyk et al. 2014). Neurons that are counted are the viable neurons with preserved nuclei. They were counted in each group for statistical quantitative analysis.

### Immunohistochemical examination

For immunohistochemical assessment of striatal GFAP, CD45 and NSE expression, the DAKO/Omnis autostainer (Agilent technologies, Denmark) was used. The automated steps included generation of labels at first, then 3 μm sections were prepared and dried at 60 °C for 20 min, followed by loading into the Omnis racks. Racks were transferred to an automated retrieval chamber and dewaxed for 30 min, followed by 30 min retrieval. Sections were pre-blocked for 5 min to prevent non-specific binding and then washed with buffer and subjected to incubation with primary antibodies against GFAP (rabbit anti-glial fibrillary acidic protein, cat. no. Z0334), CD45 (mouse anti-human CD45, leukocyte common antigen, cat. no. M0701), and NSE (mouse anti-human neuron-specific enolase, cat. no. M0873), obtained from DAKO (Agilent technologies, Denmark), for 60 min at 37 °C. A washing step was then followed by the application of secondary antibody (Dako, Copenhagen, Denmark) for 60 min, then horseradish peroxidase-conjugated streptavidin for 30 min. The 3,3′-diaminobenzidine (DAB) chromogen (Dako, Copenhagen, Denmark) was used to visualise the reaction. Hematoxylin counterstain was then applied, and the slides were mounted and inspected. GFAP, CD45 and NSE immunoreactive area percentages in individual sections were traced and computed using a Leica Microsystems image analysis system (QWin software 500MC, Germany). Five fields (x400) per slide were evaluated. Images were captured by AxioCam ERc5s Ziess camera (Zeiss, Germany).

### Statistical analysis

Initially, all results were subjected to normality testing using the Shapiro-Wilk test. Data were displayed as mean ± standard error (SEM). Normally distributed datasets were statistically analysed using unpaired Student’s two-tailed t-test and one-way analysis of variance (ANOVA) with Tukey’s post-hoc test. Non-normally distributed data were analysed by Mann Whitney or Kruskal Wallis test with Dunn’s post-hoc test. Correlations between the parameters were calculated through Spearman’s rank-order correlation analysis. All statistical comparisons were done using GraphPad Prism version 8.4.2 (GraphPad Software, USA). A probability level of < 0.05 was considered to be statistically significant.

## Results

### PTL treatment ameliorates 3NP-induced changes in the open-field test

**Fig. 2:**
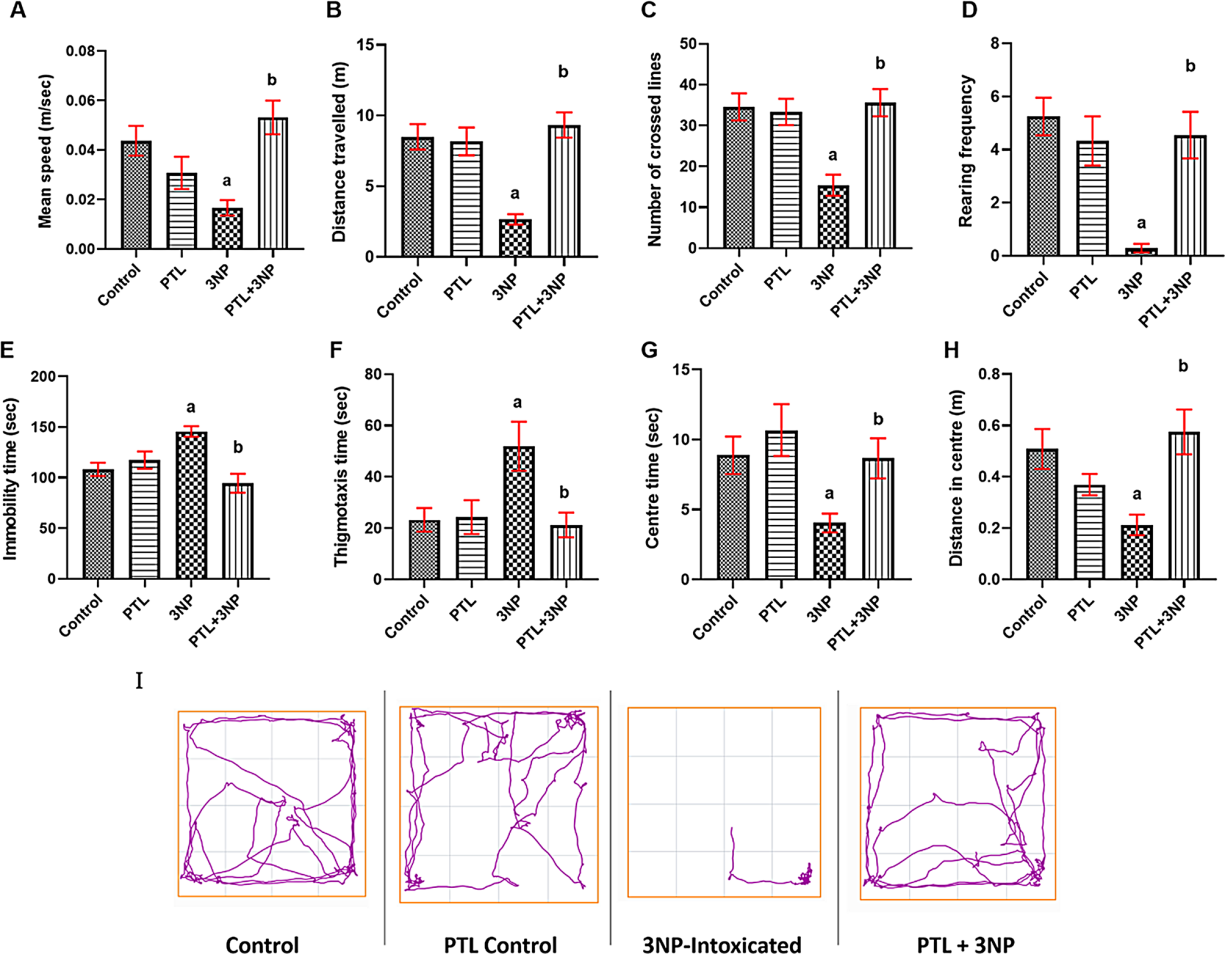
Effect of PTL on 3NP-induced neurobehavioural changes in the open-field task. (**A** – **D**) show the effect of PTL on motor activity per 3 min in the open-field, where (**A**) Mean speed, (**B**) Distance travelled, (**C**) Number of crossed lines and (**D**) Rearing frequency. (**E** – **H**) show the effect of PTL on anxious behaviour, where (**E**) Immobility time (**F**) Thigmotaxis time, (**G**) Centre time and (**H**) Distance in centre. (**I**) Track plots of rats during the open-field test. Differences among groups were analysed by one-way ANOVA followed by Tukey’s post-hoc test in (**C**), whereas statistical differences among groups were analysed by Kruskal Wallis followed by Dunn’s post-hoc test in (**A**), (**B**), (**D**), (**E**), (**G**), and (**H**). Differences among groups were analysed by Mann Whitney test in (**F**), while statistical differences between 3NP and PTL + 3NP groups were analysed by Mann Whitney in (**G**). Each column represents the mean of 10–12 rats ± SEM. a = significant difference from the control group at *P* < 0.05 and b = significant difference from the 3NP-intoxicated group at *P* < 0.05. 3NP: 3-nitropropionic acid, PTL: parthenolide, m: meter and sec: second

Behavioural and locomotor deficits are main features of experimental HD models. Herein, compared to the normal control group, continuous administration of 3NP for 14 days resulted in marked defects in motor function and anxiety-like behaviours. As for motor defects, 3NP administration significantly suppressed the mean speed (*P* = 0.0111), distance travelled (*P* = 0.0003), number of crossed lines (*P* = 0.0003) and rearing frequency (*P* < 0.0001) in the open-field task, relative to the control group. Administering PTL significantly reversed these motor deficits, as manifested by significant increments in mean speed (*P* = 0.0006), total distance travelled (*P* < 0.0001), number of crossed lines (*P* = 0.0003) and rearing frequency (*P* = 0.0005) (Fig. 2A - D).

Anxiety-like behaviours were provoked by the administration of 3NP to rats, as observed in the open-field task by significantly prolonged immobility (*P* = 0.0155) and thigmotaxis (*P* = 0.0089) times coupled with significantly decreased centre time (*P* = 0.0314) and centre distance (*P* = 0.0152), in comparison with the control animals. On the contrary, PTL treatment notably averted such perturbations where PTL-treated rats showed significantly shorter immobility (*P* = 0.0003) and thigmotaxis (*P* = 0.0079) times with concurrent marked increments in the centre time (*P* = 0.0056) and the centre distance (*P* = 0.0010) relative to the 3NP-intoxicated rats. Normal control and PTL control groups did not vary significantly in any of the behavioural variables observed in the open-field (Fig. 2E – H). Track plots of animals in the open-field supported these findings (Fig. 2I).

### PTL administration alleviates 3NP-induced changes in Morris water maze test

**Fig. 3:**
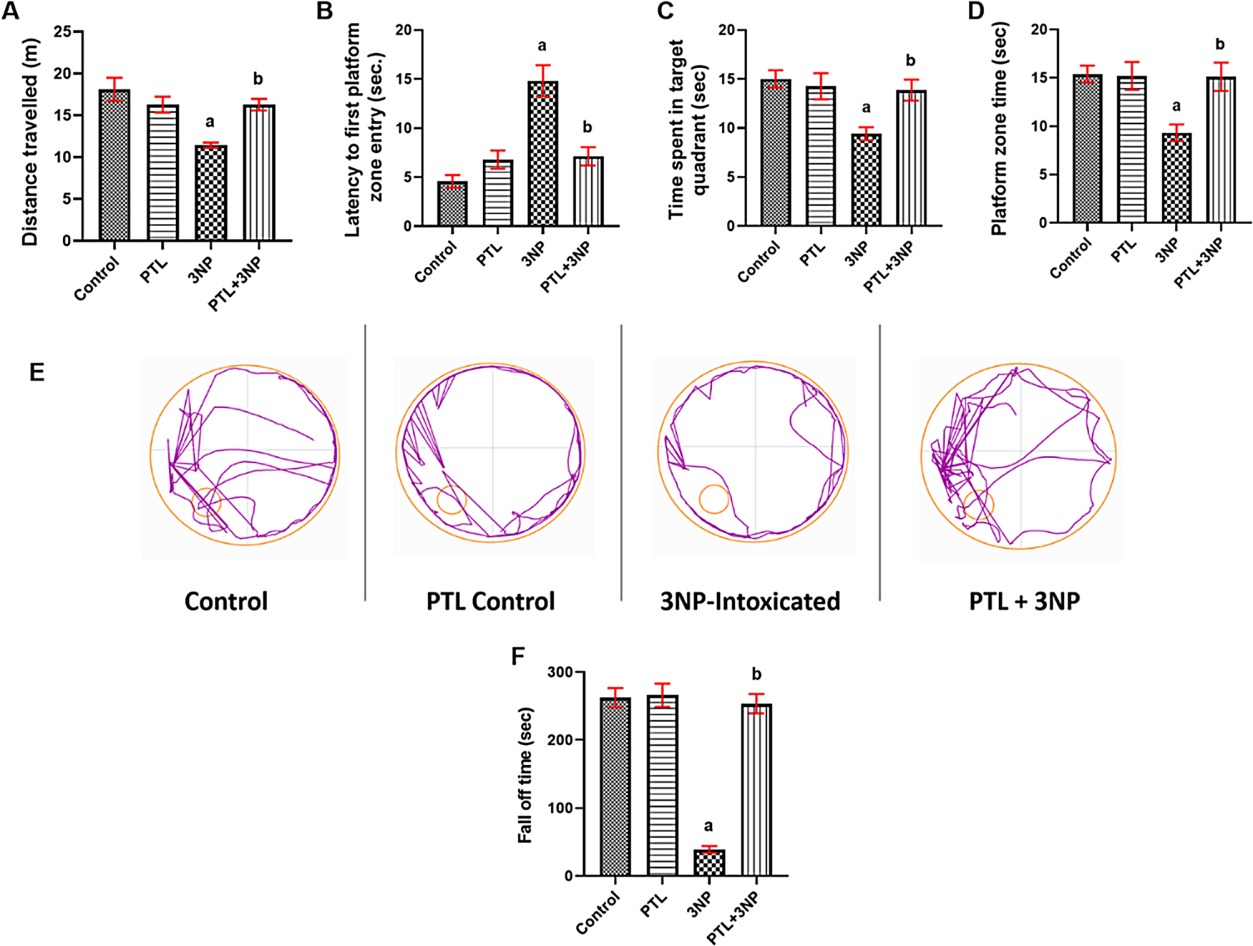
Effect of PTL on 3NP-induced neurobehavioural changes in Morris water maze and the rotarod tests. (**A**) shows the effect of PTL on motor activity in terms of distance travelled. (**B** – **D**) show the effect of PTL on spatial learning and memory where (**B**) Latency to first platform zone entry, (**C**) Time spent in target quadrant, (**D**) Platform zone time. (**E**) Track plots of rats during Morris water maze test. (**F**) Fall off time on rotarod apparatus. Differences among groups were analysed by one-way ANOVA followed by post-hoc test in (**A**) and (**B**), whereas statistical differences between groups were analysed by Kruskal Wallis followed by Dunn’s post-hoc test in (**C**), (**D**) and (**F**). Each column represents the mean of 10–12 rats ± SEM. a = significant difference from the control group at *P* < 0.05 and b = significant difference from the 3NP-intoxicated group at *P* < 0.05. 3NP: 3-nitropropionic acid, PTL: parthenolide, m: meter and sec: second

As for the defects in spatial learning and memory, 3NP administration significantly decreased the distance covered in Morris water maze test (*P* < 0.0001, Fig. 3A) and increased the latency time to first platform zone entry (*P* < 0.0001, Fig. 3B) coupled with decrease in the time spent in the target quadrant (*P* = 0.0005, Fig. 3C) and the time spent in platform zone (*P* = 0.0010, Fig. 3D) in Morris water maze task, when compared to the control rats. Treatment with PTL effectively averted these deficits and showed a marked increase in the distance travelled in Morris water maze test (*P* = 0.0014) coupled with a significant inhibition in latency time (*P* < 0.0001) and increased the duration spent in the target quadrant (*P* = 0.0036) and in platform zone (*P* = 0.0064) as compared to the 3NP group. The PTL control group displayed non-significant alterations in these behavioural activities relative to the control animals (Fig. 3A-D). Track plots of animals in Morris water maze corroborated these results (Fig. 3E).

### PTL treatment mitigates 3NP-induced alterations in the rotarod test

Deficits in locomotor coordination, muscle strength and balance were evident in the 3NP-intoxicated animals by a marked reduction in the fall-off time in the rotarod test (*P* < 0.0001) when compared to the control animals. On the other hand, PTL treatment successfully reversed these locomotor coordination deficits as the PTL-treated rats showed a significant increase in the fall-off time when compared to 3NP-intoxicated rats (*P* < 0.0001). The PTL control group did not exhibit any significant change regarding fall-off time in the rotarod test when compared to the control rats (Fig. 3F).

### PTL treatment alleviates 3NP-induced neuronal injury

**Fig. 4:**
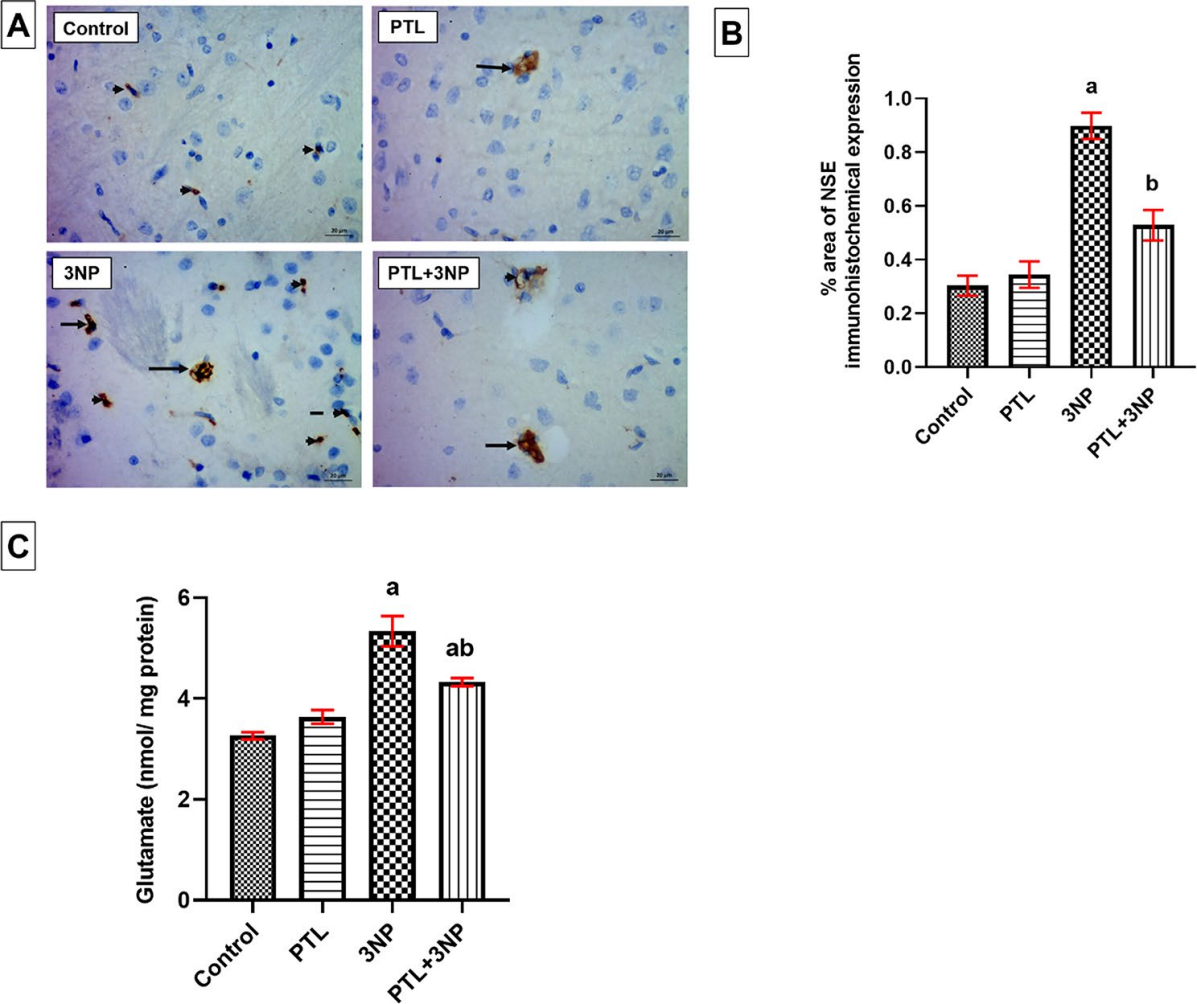
Effect of PTL on 3NP-induced neuronal injury. (**A**) Representative photomicrographs of the immunohistochemical evaluation of NSE protein expression (magnification x400). Neurons are represented by long arrows, glia by short arrows, and microglia by dashed arrows. (**B**) Area percentage of immunohistochemical expression of NSE in striatum, and (**C**) Striatal glutamate levels. Differences among groups were analysed by Kruskal Wallis followed by Dunn’s post-hoc test, while differences between 3NP-intoxicated group and PTL + 3NP group were analysed by Mann Whitney. Each column represents the mean of 6–8 rats ± SEM. a = significant difference from the control group at *P* < 0.05 and b = significant difference from the 3NP-intoxicated group at *P* < 0.05. For quantification of NSE immunostaining in the striatum, values are the mean ± SEM of the area percentage of NSE-immune staining to the total area of the microscopic field across five non-overlapping fields/section. NSE: Neuron-specific enolase. 3NP: 3-nitropropionic acid and PTL: parthenolide

3NP-intoxicated rats showed a significant increase in NSE immuno-expression (*P* = 0.0018) in striatal neurons, glia, and microglia, coupled with a marked increase in the striatal levels of the excitatory neurotransmitter, glutamate (*P* < 0.0001), when compared with the control animals. However, PTL reversed such elevations in injury markers, with significant downregulation of NSE immuno-expression (*P* = 0.0022) and glutamate levels (*P* = 0.0029). No significant differences, in terms of NSE and glutamate, were found between PTL control rats and the normal control group (Fig. 4).

### PTL administration relieves 3NP-induced striatal oxidative stress

**Fig. 5:**
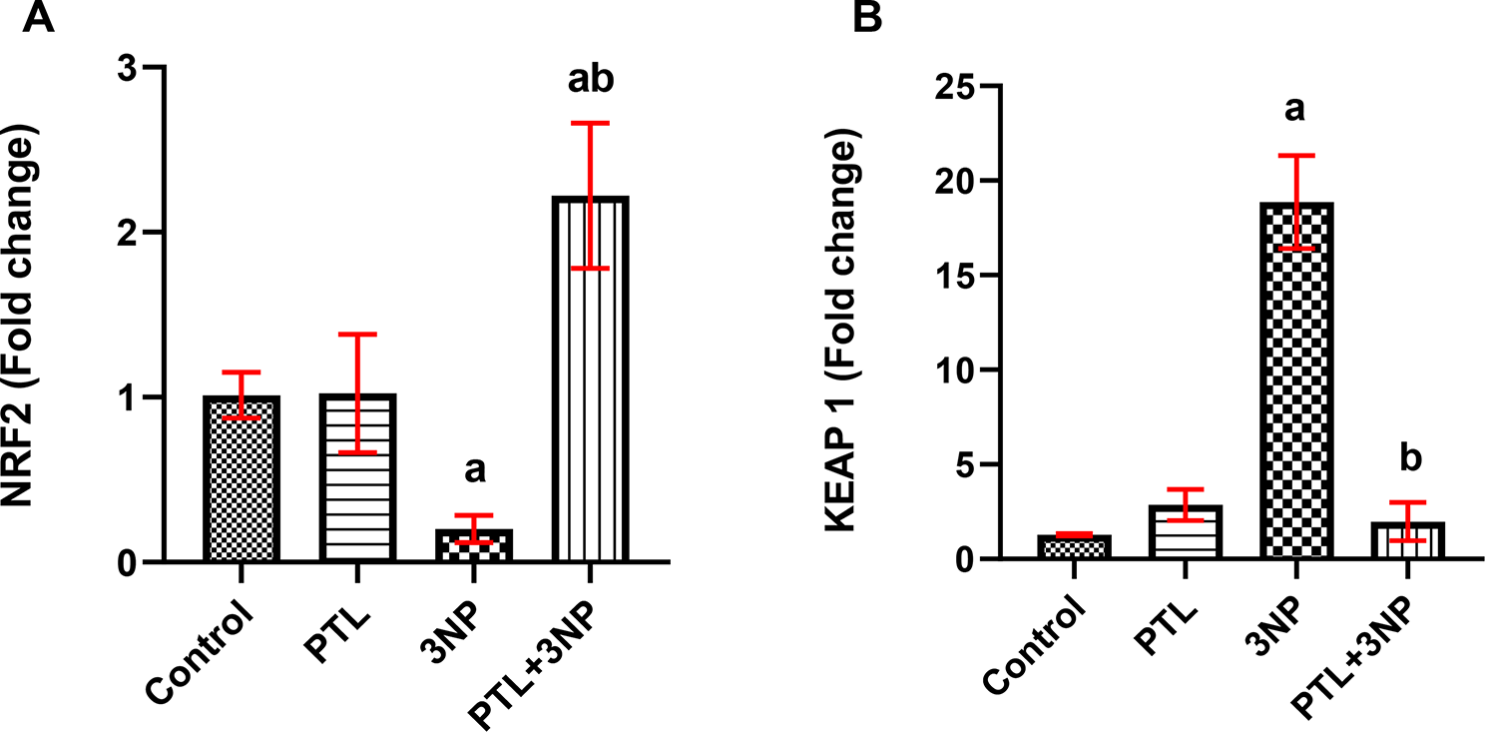
Effect of PTL on 3NP-induced changes in oxidative stress markers. Gene expression analysis of (**A**) NRF2 and (**B**) KEAP 1 in striatum. Differences among groups were analysed by Kruskal Wallis followed by Dunn’s post-hoc test, while statistical differences in NRF2 expression between control group and 3NP group were analysed by Mann Whitney. Each column represents the mean of 6–8 rats ± SEM. a = significant difference from the control group at *P* < 0.05 and b = significant difference from the 3NP-intoxicated group at *P* < 0.05. NRF2: Nuclear factor erythroid 2-related factor 2, KEAP 1: Kelch-like ECH-associated protein 1. 3NP: 3-nitropropionic acid, PTL: parthenolide

Striatal oxidative stress was observed in 3NP-injected rats where a significant suppression in striatal Nrf2 gene expression (*P* = 0.0023), coupled with increased Keap1 gene expression (*P* = 0.0316), were recorded in comparison to the control rats. As a consequence of PTL administration, remarkable recuperation in the oxidative status was observed as manifested by a significant upregulation of Nrf2 (*P* = 0.0010), accompanied by a downregulation of Keap1 expression (*P* = 0.0005) in the striatum. Both normal control and PTL control groups showed comparable oxidative status with no significant changes in any of the formerly mentioned parameters in the PTL control compared to the normal control (Fig. 5).

### PTL treatment alleviates 3NP-induced alterations in inflammasome activation

**Fig. 6:**
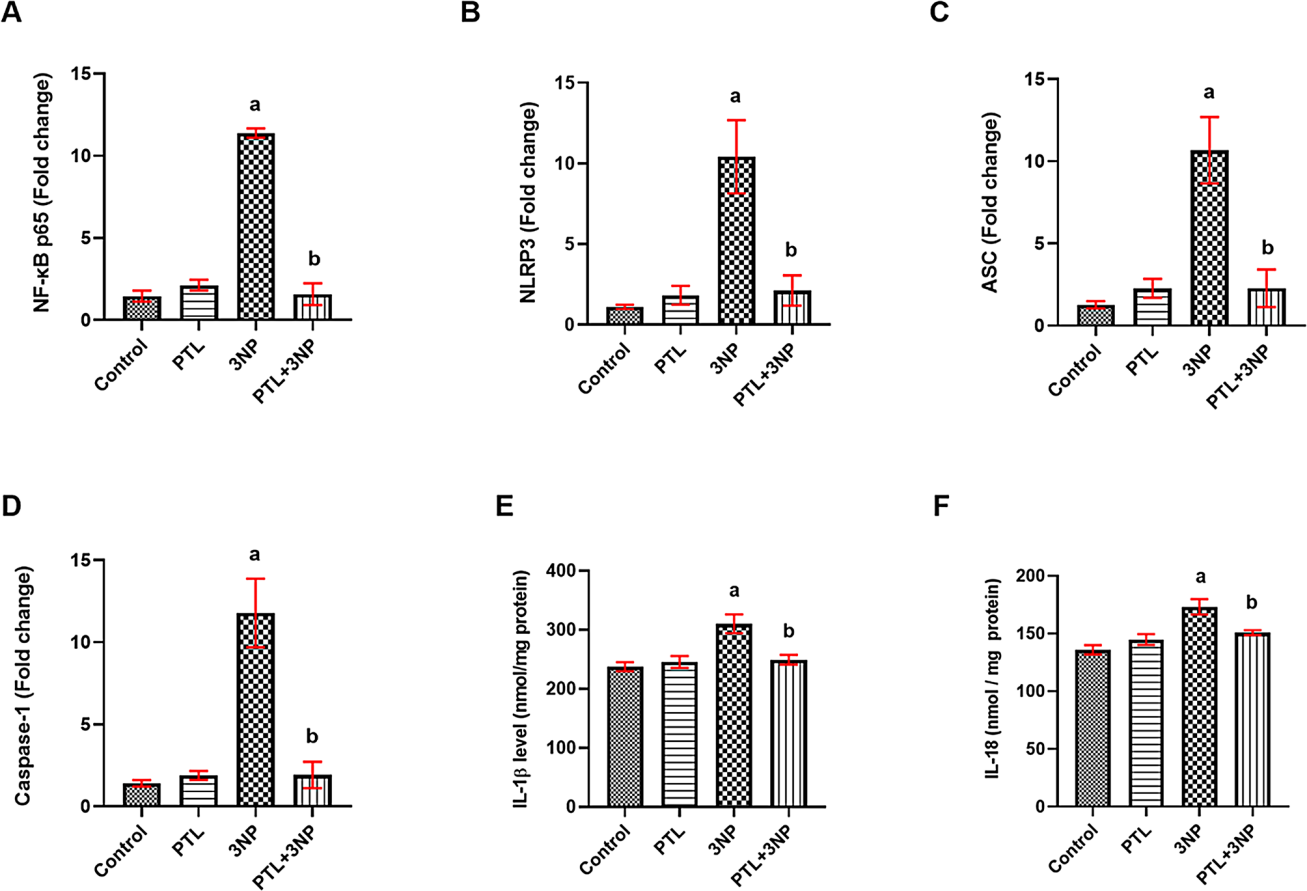
Effect of PTL on 3NP-induced changes in inflammasome activation markers. (**A** – **D**) striatal gene expression of (**A**) NF-κB p65, (**B**) NLRP3, (**C**) ASC and (**D**) Caspase-1. (**E** & **F**) show striatal levels of (**E**) IL-1β and (**F**) IL-18. Differences among groups were analysed using Kruskal Wallis followed by Dunn’s post-hoc test, Statistical differences between 3NP-intoxicated group and PTL + 3NP group were analysed by Mann Whitney in (**E**) and (**F**). Each column represents the mean of 6–8 rats ± SEM. a = significant difference from the control group at *P* < 0.05 and b = significant difference from the 3NP-intoxicated group at *P* < 0.05. NF-κB p65: Nuclear factor kappa B, NLRP3: nucleotide-binding domain leucine-rich repeat (NLR) and pyrin domain containing receptor 3, ASC: apoptosis-associated speck-like protein containing a caspase recruitment domain, IL-1β: interleukin 1 beta, IL-18: interleukin 18. 3NP: 3-nitropropionic acid and PTL: parthenolide

Rats receiving 3NP showed substantial elevations in the striatal gene expression of the p65 subunit of NF-κB (*P* = 0.0133), NLRP3 (*P* = 0.0347), ASC (*P* = 0.0175) and caspase-1 (*P* = 0.0103) along with a marked increase in the striatal levels of both IL-1β (*P* = 0.0055) and IL-18 (*P* = 0.0005), as compared with the control group. Treatment with PTL resulted in noticeable downregulation in the striatal gene expression of NF-κB p65 subunit (*P* = 0.0021), NLRP3 (*P* = 0.0068), ASC (*P* = 0.0028) and caspase-1 (*P* = 0.0021) and a significant decrease in the striatal levels of IL-1β (*P* = 0.0087) and IL-18 (*P* = 0.0012). Normal rats which received PTL did not show any significant changes in the aforementioned parameters, when compared to the normal control rats (Fig. 6).

### PTL administration ameliorates 3NP-induced microglial activation and astrocyte phenotype shifting

**Fig. 7:**
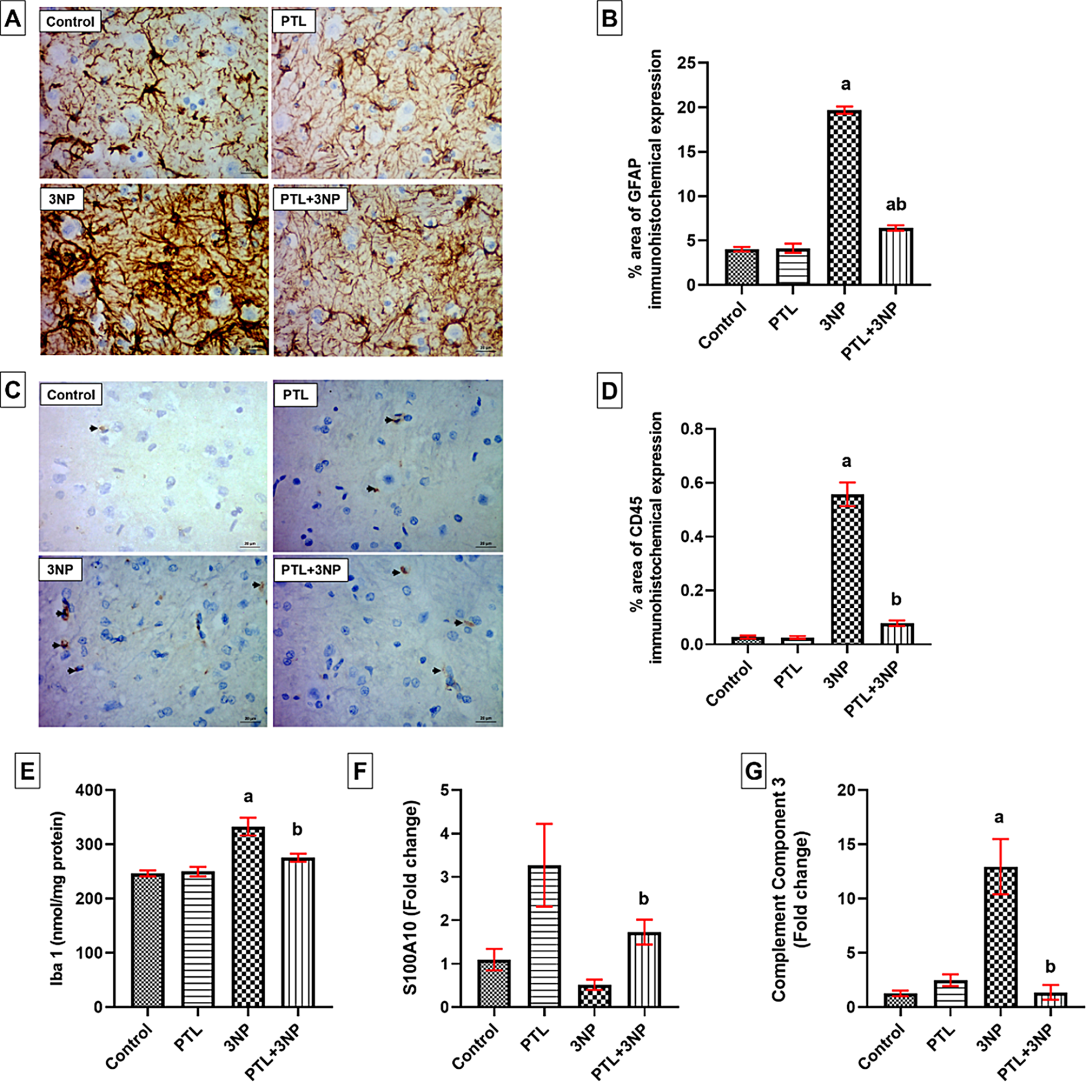
Effect of PTL on 3NP-induced microglial activation and astrocyte phenotype shifting. (**A**) Representative photomicrographs of the immunohistochemical evaluation of GFAP protein expression (magnification x400) and (**B**) Area percentage of immunohistochemical expression of GFAP in striatum (**C**) Representative photomicrographs of the immunohistochemical evaluation of CD45 protein expression (magnification x400) and (**D**) Area percentage of immunohistochemical expression of CD45 in striatum. (**E**) Striatal Iba-1 concentrations. (**F** & **G**) Gene expression analysis of striatal S100A10 (**F**) and complement component-3 (**G**). Differences among groups were analysed by one-way ANOVA followed by Tukey’s post-hoc test in (**B**), (**D**) and (**E**), whereas statistical differences among groups were analysed using Kruskal Wallis test followed by Dunn’s post-hoc test in (**F**) and (**G**). Each column represents the mean of 6–8 rats ± SEM. a = significant difference from the control group at *P* < 0.05 and b = significant difference from the 3NP-intoxicated group at *P* < 0.05. For quantification of GFAP and CD45 immunostaining in the striatum, values are the mean ± SEM of the area percentage of GFAP-immune staining and CD45-immune staining, each separately, to the total area of the microscopic field across five non-overlapping fields/section. GFAP: glial fibrillary acidic protein, CD45: cluster of differentiation 45, Iba-1: ionized calcium-binding adapter molecule 1, S100A10: S100 calcium-binding protein A10. 3NP: 3-nitropropionic acid and PTL: parthenolide

Administration of 3NP provoked microglial and astroglial activation directing it towards the neurotoxic A1 phenotype. The immunohistochemical examination of striatal sections demonstrated that the immuno-expression of striatal CD45 was significantly higher among rats that received 3NP when compared to the control rats (*P* < 0.0001). Moreover, 3NP-intoxicated rats showed a significant elevation in striatal Iba1 level (*P* < 0.0001) compared to the control rats. However, PTL-treated rats showed a significant decrease in striatal CD45 immuno-expression where minimal unremarkable staining of CD45 was observed (*P* < 0.0001) with a concurrent decrease in striatal Iba1 level (*P* = 0.0038). The PTL control group showed insignificant changes in CD45 immunostaining and Iba1 striatal level compared to the control animals (Fig. 7).

As for striatal GFAP immunoreactivity, control rats showed normal astroglial distribution and normal glial fibrillary processes. On the other hand, striatal sections from 3NP-intoxicated rats revealed a remarkable astroglial proliferation reflected as a significant increase in striatal GFAP immuno-expression (*P* < 0.0001) in response to tissue injury. On the contrary, PTL-treated rats showed mild astroglial proliferation that can be interpreted as a sign of striatal regeneration accompanied with a significant decrease in striatal GFAP immuno-expression (*P* < 0.0001) when compared to the 3NP group. The PTL control group did not exhibit any abnormal changes regarding GFAP immunohistochemical expression when compared to the control rats (Fig. 7).

3NP-injected rats showed a statistically non-significant decrease in striatal gene expression of S100A10 (*P* = 0.7717) accompanied with a significant increase in striatal gene expression of complement component 3 (*P* = 0.0088), as compared to the control animals. This phenotypic transformation from astrocyte A2 to astrocyte A1 was reversed in rats treated with PTL as shown by a marked increase in striatal gene expression of S100A10 (*P* = 0.0260) and a significant decrease in C3 gene expression in striatum (*P* = 0.0017). The PTL control rats showed an apparent increase in striatal gene expression of S100A10 relative to the control group, yet the difference did not reach statistical significance (*P* = 0.2956), without any significant change in the striatal gene expression of C3 (Fig. 7).

### PTL treatment mitigates 3NP-induced histological alterations in striatum

**Fig. 8:**
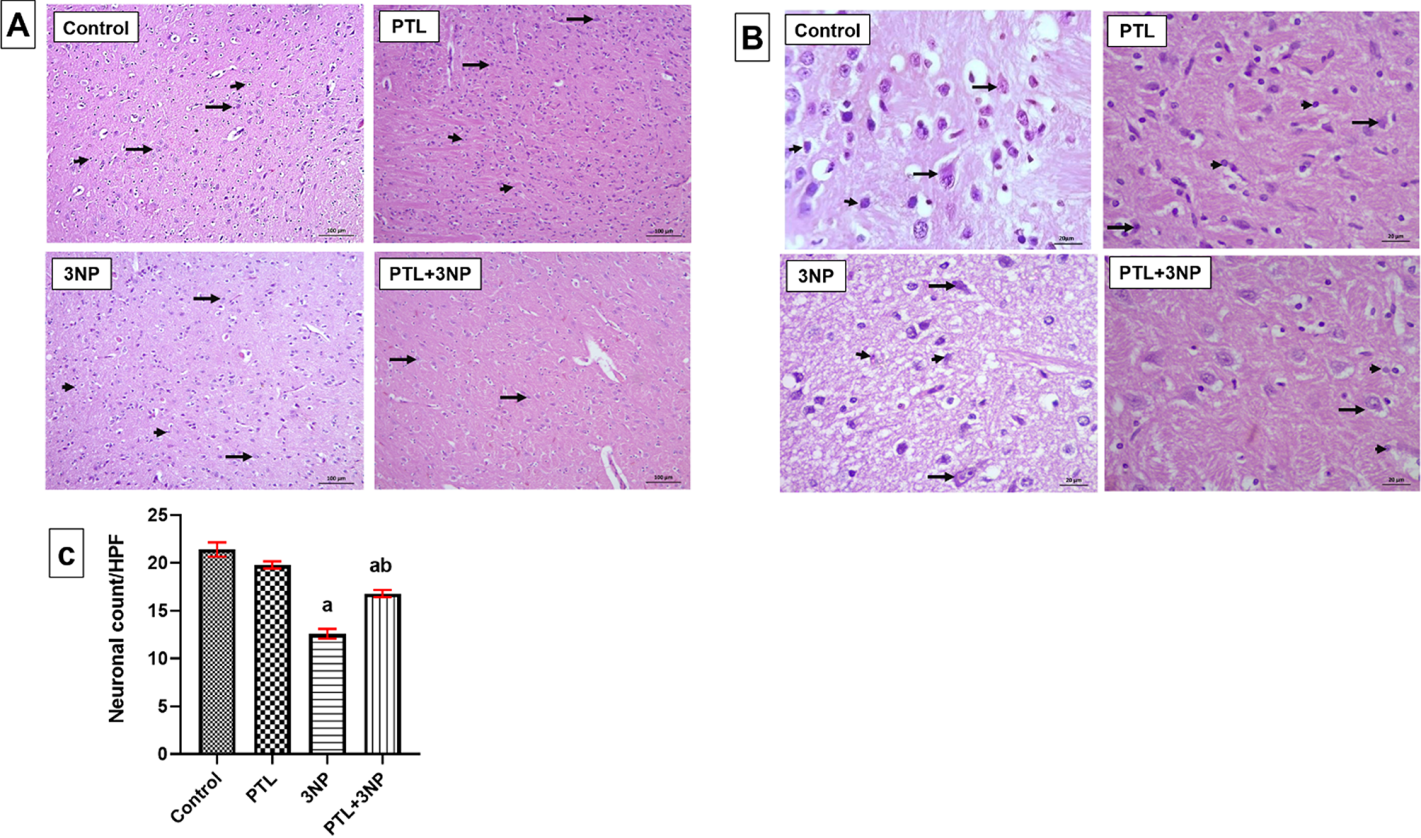
H&E-stained striatal sections showing effect of PTL on 3NP-induced histopathological alterations. (**A**) Representative photomicrographs of histological examination (magnification x100) showing control group with preserved neuronal structures (long arrows) and normal glia (short arrows), whereas in 3NP group neuronal damage (long arrows) and proliferating astroglia (short arrows) was evident. PTL control group showed preserved neuronal structures (long arrows) and unremarkable glia (short arrows) where PTL treatment group presented with fairly preserved neuronal structures (long arrows) and mild astroglial proliferation (short arrows). (**B**) Representative photomicrographs of histological examination (magnification x400), showing control group with preserved neuronal structures (long arrows) and normal glia (short arrows), while 3NP group showed neuronal damage with shrunken neurons (long arrows), and proliferating astroglia (short arrows). Finally, PTL control group showed preserved neuronal structures (long arrows) and normal glia (short arrows) whereas the PTL treatment group presented with fairly preserved neuronal structures (long arrows) and mild astroglial proliferation (short arrows). (**C**) Neuronal count per high power field of H&E-stained striatal brain sections. Differences among groups were analysed by one-way ANOVA followed by Tukey’s post-hoc test. Each column represents the mean of five non-overlapping fields per slide counted in high power field of H&E-stained striatal brain sections of 6 rats per group. a = significant difference from the control group at *P* < 0.05 and b = significant difference from the 3NP-intoxicated group at *P* < 0.05. H&E: haematoxylin and eosin, HPF: high power field, 3NP: 3-nitropropionic acid and PTL: parthenolide

Hematoxylin and eosin-stained tissue sections from the different experimental groups were inspected histopathologically. Photomicrographs of the control group showed preserved neuronal structures and normal glia, whereas the 3NP group showed extensive neuronal damage, with proliferating astroglia and vacuolated shrunken neurons. Treatment with PTL induced a significant neuroprotection where the neuronal structures were fairly preserved with only mild astroglial proliferation. PTL control rats were histologically comparable to control rats and showed almost the same records without abnormal histological changes (Fig. 8).

### Correlation of astrocyte and microglial markers with the studied parameters

**Fig. 9:**
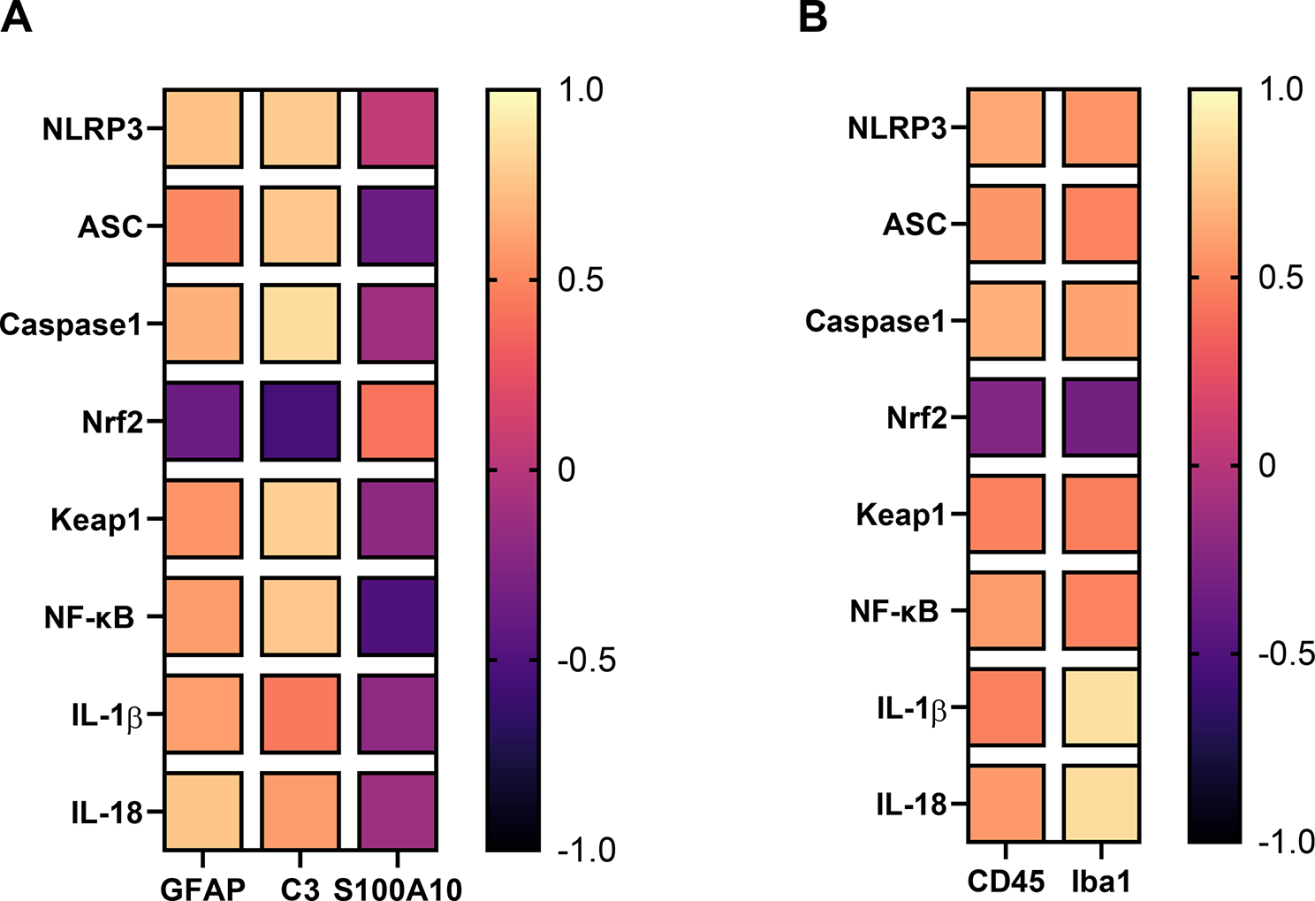
Heatmaps summarizing the correlations of astrocyte and microglial markers with the studied parameters. (**A**) Heatmap for the correlation between astrocyte markers and the studied parameters. (**B**) Heatmap for the correlation between microglial markers and the studied parameters. Correlations were analyzed by Spearman correlation and Spearman correlation coefficients (r) between the markers were plotted in the heatmap. The dark-blue colour corresponds to a correlation coefficient close to -1 and the light-yellow colour corresponds to values close to 1. NLRP3; nucleotide-binding domain leucine-rich repeat (NLR) and pyrin domain containing receptor, ASC; apoptosis-associated speck-like protein containing a caspase recruitment domain, Caspase-1, NF-κB; Nuclear factor kappa B, Nrf2; Nuclear factor erythroid 2-related factor 2, Keap1; Kelch-like ECH-associated protein 1, IL-1β; interleukin 1 beta, IL-18; interleukin 18, GFAP; glial fibrillary acidic protein, C3; complement component 3, S100A10; S100 calcium-binding protein A10, CD45; cluster of differentiation 45, Iba1; ionized calcium-binding adapter molecule 1

A correlation analysis was performed between the astrocyte markers (GFAP, C3 and S100A10) and the following markers: NLRP3, ASC, caspase-1, NF-κB, Nrf2, Keap1, IL-1β, and IL-18, in addition to another correlation analysis that was performed between the measured microglial markers (CD45 and Iba1) and the same set of parameters. Several significant positive correlations were found between the astrocyte markers and the measured parameters as shown between C3 and caspase-1 (*r* = 0.86, *p* = 1.1979E-08), C3 and Keap1 (*r* = 0.8, *p* = 5.04582E-07), GFAP and IL-18 (*r* = 0.76, *p* = 1.70168E-05), GFAP and NLRP3 (*r* = 0.74, *p* = 3.13922E-05), among many others shown in Fig. 9A and Supplementary Table 1. Also, significant negative correlations were observed between C3 and Nrf2 (*r*=-0.56, *p* = 0.003950972) as well as between S100A10 and NF-κB (*r*=-0.51, *p* = 0.009450281) as depicted in Fig. 9A and Supplementary Table 1.

Correlation analysis also detected significant positive correlations between the microglial markers and the measured parameters as manifested between Iba1 and IL-1β (*r* = 0.87, *p* = 6.0047E-09), Iba1 and IL-18 (*r* = 0.85, *p* = 1.6874E-08), CD45 and caspase-1 (*r* = 0.67, *p* = 0.00036839), among many others shown in Fig. 9B and Supplementary Table 2.

## Discussion

The current study provides the first evidence of the neuroprotective effect of PTL against 3NP-induced neurotoxicity. Systemic administration of the mitochondrial toxin, 3NP, to rats provides a reliable experimental model that closely simulates the behavioural abnormalities, biochemical manifestations, and pathophysiological characteristics of HD in humans (Túnez et al. 2010). In this study, the NF-κB inhibitor, PTL, managed to improve the neurobehavioural defects inflicted by 3NP on rats, relieve oxidative stress, alleviate neuronal injury and microglial activation, induce astrocyte shifting towards the neuroprotective astrocyte A2 phenotype, reduce inflammasome activation and finally reverse 3NP-induced histopathological alterations.

The striatum is the main input structure to the information-processing centre in the basal ganglia which executes and organizes the behavioural output after receiving inputs from the cognitive and motor cortical regions (Haber 2003). The striatum is by far the most affected brain region in HD patients (Nopoulos 2016; Yang et al. 2021). As depicted in our study, systemic administration of 3NP significantly induced HD-like manifestations, demonstrated by impairment in the motor activity, locomotor coordination, spatial learning, and memory function in addition to increased anxiety-like behaviour. Our findings concur with various former studies reporting impaired locomotor activity after 3NP systemic administration (Chakraborty et al. 2014; Sayed et al. 2020; Shalaby et al. 2018). Such motor derangement has been linked to the 3NP-induced neuronal death in the striatum and cortex, as those regions are responsible for controlling body movement (Courtes et al. 2015), where reduced numbers of morphologically normal granule cells were found in 3NP-intoxicated animals (Chakraborty et al. 2014). Moreover, significant increase in anxiety-like behaviour in addition to deterioration in spatial learning and memory function was also reported in several earlier studies adopting the 3NP animal model (Chakraborty et al. 2014; Ibrahim and Abdel Rasheed 2022; Jamwal and Kumar 2017; Sayed et al. 2020; Shalaby et al. 2018). Abnormalities in behavioural and motor activities could be indicative of striatal degeneration induced by 3NP-intoxication (Vis et al. 1999), which might be attributed to the excessive generation of ROS and free radicals in addition to increased brain protein oxidation (Ahmed et al. 2016; Forster et al. 1996).

In the present study, PTL treatment significantly reversed the 3NP-induced perturbations in motor activity, locomotor coordination, spatial learning, memory function, and averted anxiety-like behaviour in rats. In this context, PTL has previously improved locomotor activity, spatial learning and memory function in addition to ameliorating anxiety-like behaviours in rat models of diabetes-induced neuropathy and TBI (Ding et al. 2022; Galeotti et al. 2014; Khare et al. 2017). Cognitive functional recovery observed in PTL-treated animals can be attributed to the pharmacological inhibition of NF-κB signaling pathway (Khare et al. 2017). Earlier reports have also described an anti-serotonergic potential for PTL which can explain its anxiolytic effect (Nawrot et al. 2019; Weber et al. 1997).

Oxidative stress is a key player in many neurodegenerative disorders (Niedzielska et al. 2016). Nrf2/Keap1 pathway is a widely known antioxidant defence mechanism, where Nrf2 is an antioxidant transcription factor, responsible for removing excess ROS, that is naturally repressed by Keap1 (Abdelmonem et al. 2024; Alfieri et al. 2011). Several reports have demonstrated how Nrf2 activation, through its release from binding to Keap1 protein, protects against oxidative stress in both in vitro and in vivo models of neurodegenerative disease (Brandes and Gray 2020; Dinkova-Kostova et al. 2018), including 3NP-induced HD’s like disease in rats (Gonchar et al. 2021).

Cytoplasmic Nrf2 levels are normally maintained low by its coupling with Keap1 which targets Nrf2 ubiquitination/proteasomal degradation. Under conditions of oxidative stress and exposure to Keap1-modifing agents, the Keap1/Nrf2 interaction is altered with consequent liberation of Nrf2. Nuclear translocation of Nrf2 then takes place to trigger the transcription of a multitude of antioxidant-related genes (Habib et al. 2022; Kobayashi et al. 2004). The present investigation revealed that administration of 3NP, which is a potent suicide inhibitor for SDH, complex II, was associated with increased oxidative stress and repressed antioxidant defence, evidenced by the downregulation of Nrf2 and a simultaneous upregulation of Keap1 striatal expressions. Our findings coincide with several earlier reports of 3NP-induced HD-like disease showing decreased striatal expression of Nrf2 at the gene and protein levels (Gendy et al. 2023; Ibrahim and Abdel Rasheed 2022; Mustafa et al. 2021). The oxidative stress observed herein and in earlier reports can be explained by the 3NP-induced irreversible SDH inhibition, which in turn disrupted the electron transport chain (ETC), caused substantial ATP depletion and eventually resulted in neuronal death (Túnez et al. 2010).

PTL administration in the present study alleviated 3NP-induced oxidative stress with significant enhancement in Nrf2 expression, whereas Keap1 expression was noticeably reduced. Our results align with earlier studies showing that PTL activates the Nrf2/Keap1 axis through elevating Nrf2 and suppressing Keap1 protein contents in adipocytes in an obesity-induced inflammatory response model (Kim et al. 2019), and in an adipocyte differentiation model (Kim et al. 2020). In addition, PTL was reported to incite significant Nrf2 upregulation in a radiation-induced CNS injury mouse model (Jinling et al. 2022). The antioxidant effect of PTL was previously demonstrated in normal prostate epithelial cells, where PTL increased the oxidation of Keap1 leading to increased Nrf2 levels and subsequent Nrf2-dependent expression of different antioxidant enzymes (Xu et al. 2013).

Nrf2 signaling has been previously implicated in neuroprotection via inhibiting the redox-sensitive transcription factor NF-κB. Oxidative stress is known to activate the classical proinflammatory transcription factor, NF-κB, and can be counteracted by the Nrf2-dependent upregulation of target antioxidant genes (Brandes and Gray 2020). Additionally, Nrf2 has been reported to block tumor necrosis factor-α (TNF-α)-induced activation of NF-κB and subsequent nuclear translocation (Bellezza et al. 2012). In the current study, 3NP significantly upregulated the expression of NF-κB p65 subunit. The low Nrf2 levels reported herein following 3NP-intoxication may be a factor contributing to the increased NF-κB p65 gene expression, These results are corroborated by several studies in HD-like rat model (Gendy et al. 2023; Ibrahim and Abdel Rasheed 2022; Jang and Cho 2016; Mansour et al. 2022). Activation of NF-κB in microglia and astrocytes mediates the production of enormous quantities of proinflammatory cytokines such as TNF-α, IL-1β and IL-6, as well as ROS and excitotoxins (Mattson and Meffert 2006), which collectively mediate tissue damage and neuronal death (Bai et al. 2019; Brandes and Gray 2020).

In the current study, NF-κB p65 expression was downregulated upon administration of PTL. PTL is a well-known NF-κB inhibitor and has shown downregulation of NF-κB level in a type 2 diabetes rat model (Khare et al. 2017), and in a TBI mouse model (Ding et al. 2022). Such results can be explained by the fact that PTL binds directly and inhibits IκB kinase (IKK) enzyme, responsible for nuclear translocation of NF-κB (Hehner et al. 1999a; Mathema et al. 2012; Nam 2006; Zhu et al. 2023). In unstressed cells, NF-κB interacts with its inhibitor IκB causing its retention in the cytoplasm and then, as a result of various proinflammatory stimuli, IκB is subjected to IKK-mediated phosphorylation which triggers its ubiquitination with consequent proteasome-mediated degradation, enabling NF-κB to move from the cytoplasm to the nucleus and exert its inflammatory action (Kwok et al. 2001; Zinatizadeh et al. 2021). Another proposed mechanism for PTL-mediated IKK inhibition is via preventing TNF-α-induced activation of IKK which will eventually hamper NF-κB nuclear translocation (Hehner et al. 1999b; Zhu et al. 2023).

The upregulation of NF-κB contributes to the activation and assembly of NLRP3 inflammasome (Shi et al. 2016). Activated NLRP3 inflammasome is responsible for the cleavage and activation of caspase-1 with consequent release of IL-1β and IL-18 leading to severe inflammatory reactions and pyroptosis-induced neuronal death (Cui et al. 2020).

In our study, 3NP-intoxicated rats showed inflammasome activation similar to that shown in HD and its transgenic animal model (Chen et al. 2022; Voet et al. 2019). Our results demonstrated a marked increase in the striatal expression of NLRP3, ASC, caspase-1 and a significant increase in the striatal contents of IL-1β and IL-18 were shown. The results reported herein are in line with earlier studies reporting 3NP-induced NLRP3 inflammasome activation and subsequent neuroinflammation (Abdel Rasheed and Ibrahim 2022; Jamwal and Kumar 2016; Mansour et al. 2022). Such results can be explained in the light of our aforementioned results revealing that 3NP activated the expression of NF-κB p65.

In the current study, our data revealed that PTL administration suppressed the NLRP3 inflammasome and its ensuing inflammatory events by decreasing the striatal gene expression of NLRP3, ASC and caspase-1 and thereby diminishing striatal IL-1β and IL-18 levels. Inhibition of NLRP3 inflammasome activation after PTL treatment was previously reported in a TBI rat model (Ding et al. 2022), in a lipopolysaccharide (LPS)-induced AD mouse model (Fan et al. 2023) and in obesity-induced inflammatory response in a mouse model (Kim et al. 2019). PTL has been shown to inhibit the oligomerization of NLRP3, increase the NLRP3 degradation (Fan et al. 2023; Juliana et al. 2010; Kim et al. 2019; Liu et al. 2023), and block the NLRP3-ASC interaction (Liu et al. 2023).

Another contributor to neuronal cell damage and death is the excitatory neurotransmitter, glutamate, owing to its role in neuronal hyper-excitability (Khadrawyb YA 2014; Lau and Tymianski 2010; Pál 2018). Moreover, NSE has been used as an efficient valuable marker of neuronal injury, cell death, neuroinflammation and neuronal integrity (Haque et al. 2018). In the current investigation, 3NP-intoxicated rats showed a significant increase in striatal glutamate level and in NSE immunohistochemical expression. Disturbances in neurotransmission have been extensively studied and linked to HD pathogenesis (Suganya and Sumathi 2017). Many symptoms in HD patients are linked to aberrant neurotransmission caused by the imbalance between excitatory and inhibitory neurotransmitters, such as glutamate and GABA, respectively. In fact, our results agree with numerous studies showing alteration in neurotransmitters and elevation of striatal glutamate levels following 3NP injection (Abdelfattah et al. 2020; Jamwal and Kumar 2017; Sayed et al. 2020; Shalaby et al. 2018). The oxidative stress-induced mitochondrial dysfunction may provide a valid justification for the neuronal excitotoxicity caused by glutamate in 3NP-intoxicated rats (Sayed et al. 2020). Furthermore, ROS generated in response to 3NP intoxication are known to be associated with activation of glutamate receptors and excessive glutamate production (Kolodziejczyk et al. 2010; Abdelfattah et al. 2020).

As for our reported NSE data, such results are in line with several studies reporting an elevation in NSE immunohistochemical expression after 3NP exposure in mice (Virdi et al. 2021). NSE, which is involved in neuronal glycolysis, has been considered as a direct biomarker to assess functional neuronal damage in several neuronal pathologies including TBI, HD-like rat model and experimental epilepsy model (Baraka et al. 2012; Ramirez et al. 2021; Virdi et al. 2021).

PTL is known to possess neuroprotective activity which has been previously evidenced in LPS-induced AD mouse model (Fan et al. 2023), and in TBI mouse model (Ding et al. 2022). Nonetheless, the effect of PTL on preserving neuronal glutamate and NSE has not yet been evaluated. Herein, PTL treatment achieved notable neuroprotection that was evidenced by a marked decline in striatal glutamate level and NSE immunohistochemical expression suggesting a neuronal preserving effect for PTL against 3NP-induced neurotoxicity.

Microglial activation and proliferation are fundamental to neuroinflammation. An earlier investigation suggests that a key event in HD pathogenesis is the activation of microglial cells (Sapp et al. 2001). Microglia are activated in several neurological diseases including HD and Parkinson’s disease (PD), where the activated microglia are believed to release proinflammatory cytokines (Block and Hong 2007; Paldino et al. 2020; Yang et al. 2017). Herein, activated microglial cells were detected by the microglial marker Iba1, a calcium-binding protein found on the surface of microglia (Jurga et al. 2020; Liao et al. 2020; Wang et al. 2022), in addition to CD45 which is expressed by both resting and activated microglia (Honarpisheh et al. 2020; Korzhevskii and Kirik 2016).

Our data revealed that 3NP boosted the striatal level of Iba1 and the striatal immunohistochemical expression of CD45 reflecting the increased presence of activated microglial cells and such activated microglia could contribute to the altered expression and secretion of the pro-inflammatory mediators, NF-κB and IL-1β. These results can be corroborated by several earlier studies that showed microglial activation, confirmed by a significant increase in CD45 and Iba1 immunohistochemical expression in 3NP-intoxicated rat brains (Bak et al. 2016; Jang and Cho 2016; Mansour et al. 2022; McBride et al. 2003). The herein reported microgliosis might be explicated based on the noted NF-κB activation, which is known to induce microglial activation eventually promoting cell death in rat models of 3NP (Ali et al. 2021; Gendy et al. 2023; Ibrahim and Abdel Rasheed 2022; Mémet 2006).

In the present study, after treatment with PTL, we detected the repression of these microglial markers, Iba1 and CD45. The current results agree with previous findings where PTL curbed Iba1 levels in activated microglia in a LPS-induced AD mouse model (Fan et al. 2023), and in a TBI rat model (Ding et al. 2022). Such results can be explained in the light of the previously reported ability of PTL to suppress microgliosis and inhibit microglial-mediated neuroinflammation, evidenced by the decreased number of migrated microglia and the restoration of microglial phagocytic activity (Fan et al. 2023; Gaojian et al. 2020). We also can hypothesize that the inhibitory effect of PTL over NF-κB activation, reported earlier in this study, can also contribute to the microglial repression, evidenced by our Iba1 and CD45 results.

The present investigation showed an upsurge in the immunohistochemical expression of GFAP following 3NP administration which is in line with previous reports demonstrating elevation of striatal GFAP immunohistochemical expression in response to 3NP systemic administration in HD rat models (Elbaz et al. 2019; Ibrahim and Abdel Rasheed 2022). In fact, increased striatal expression of GFAP, which is used to indicate the magnitude of astrocyte activation, is considered a valid sign of neurodegeneration. Moreover, the elevation of GFAP following striatal neuronal injury and astrogliosis has been observed as a long-standing pathological feature in the 3NP model of HD (Abdelfattah et al. 2020).

Interestingly in our study, the astrocyte activation instigated by 3NP was mitigated upon PTL administration. The current results indicated that PTL treatment has an anti-inflammatory effect and can suppress astrogliosis, which is in line with the findings of several previous studies where PTL reduced hippocampal GFAP expression in mouse models of LPS-induced AD (Fan et al. 2023) and spinal cord injury (Gaojian et al. 2020), and decreased GFAP-positive cells found in the spinal cord and brain of a collagen antibody-induced arthritis mouse model (Williams et al. 2020).

Astrocytes have gained much attention due to their contribution in the neuroinflammation witnessed in neurodegenerative pathologies (Li et al. 2019; Neal and Richardson 2018; Palpagama et al. 2019). The neurotoxic A1 astrocytes are reported to be abundant in a number of human neurodegenerative illnesses including HD, AD and PD (Brandebura et al. 2023; Simpson et al. 2010). In the current study, 3NP administration dramatically induced A1 astrocytes, as manifested by the increased C3 expression, with a concurrent decrease in A2 astrocyte-related gene S100A10 expression. These results are supported by other studies showing an elevation in C3 expression in the striatum and hippocampus in the 3NP-induced HD-like rat model (Lopez-Sanchez et al. 2020, 2022) indicating an active shift of astrocytes towards the A1 neurotoxic phenotype.

Our results showed that PTL treatment reversed these changes by inducing neuroprotective A2 astrocytes and increasing the expression of their marker, S100A10, coupled with a decrease in A1 astrocyte activity and C3 expression. These results agree with an earlier report where PTL attenuated the activation of A1 astrocytes through decreasing the C3 immunofluorescence staining in spinal cord tissues in a mouse model used to study spinal cord injury (Gaojian et al. 2020). Collectively, the observations reported herein can provide a new mechanistic framework to better understand the neuroprotective effects of PTL on 3NP intoxication, where PTL has managed to reverse the astrocyte shift back to the neuroprotective A2 phenotype.

Neuroinflammation in HD is associated with striatal degeneration and extensive neuronal loss that represent characteristic features of HD progression (Nopoulos 2016). Histopathological examination in the present study revealed striatal neuronal damage and proliferating astroglia after 3NP systemic administration. Moreover, 3NP administration decreased the neuronal count as detected in the H&E-stained striatal brain sections. These results are consistent with several previous reports that demonstrated striatal neuronal death in the 3NP-induced HD-like rat model (Abdelfattah et al. 2020; Chakraborty et al. 2014; Gao et al. 2015; Ibrahim and Abdel Rasheed 2022). Striatal neuronal death may be attributed to several mechanisms. The overproduction of pro-inflammatory cytokines, as a result of activated inflammatory cascades, enhances neuronal death (Abdelfattah et al. 2020; Gao et al. 2015). Neuronal death is also associated with oxidative stress progression, mitochondrial impairments and ROS production (Almeer et al. 2019). Therefore, the present histological aberrations are in harmony with the oxidative and inflammatory perturbations witnessed in the currently studied model.

In support of our observed neuroprotective proficiencies of PTL, 3NP-induced histopathological abnormalities were mitigated upon PTL administration. PTL alleviated neuronal death as portrayed by fairly preserved neuronal structures with only mild astroglial proliferation in the striata of rats along with significant increase in striatal neuronal count as compared with the 3NP-treated rats. Our results coincide with other studies demonstrating the neuroprotective effect of PTL against microglia-mediated neuronal cell death in a LPS-induced AD mouse model (Fan et al. 2023). Additionally, PTL was previously reported to alleviate neuronal apoptosis and neurological damage via decreasing the expression of inflammatory factors, including IL-6 and TNF-α and increasing the expression of antioxidant GSH, in addition to changing the polarization of microglia in a TBI mouse model (Ding et al. 2022).

Finally, this study has some limitations that should be acknowledged. First, this study is an in vivo study, based on a rat model, using the mitochondrial toxin, 3NP, which has been shown to mimic HD and has been used as a model for HD. Therefore, we urge future studies to confirm the effects of PTL in transgenic HD models. Regarding the effect of PTL on neuronal, glial and microglial cell count, a more comprehensive analyses should be performed using immunofluorescent techniques and more specific neuronal markers such as neuron-specific nuclear binding protein (NeuN). Moreover, additional in vitro studies are also recommended to investigate the effect of PTL on different neuronal cell lines and to assess its effect on the activation of inflammasome pathway in isolated primary cultures of microglia and astrocytes. Furthermore, investigating the effect of PTL on 3NP-induced HD-like symptoms in female animals could shed light on the potential influence of gender on PTL-induced neuroprotection in this model. Our study and the recommended prospective studies may pave the way for further clinical evaluation of PTL as a promising anti-HD drug.

**Fig. 10:**
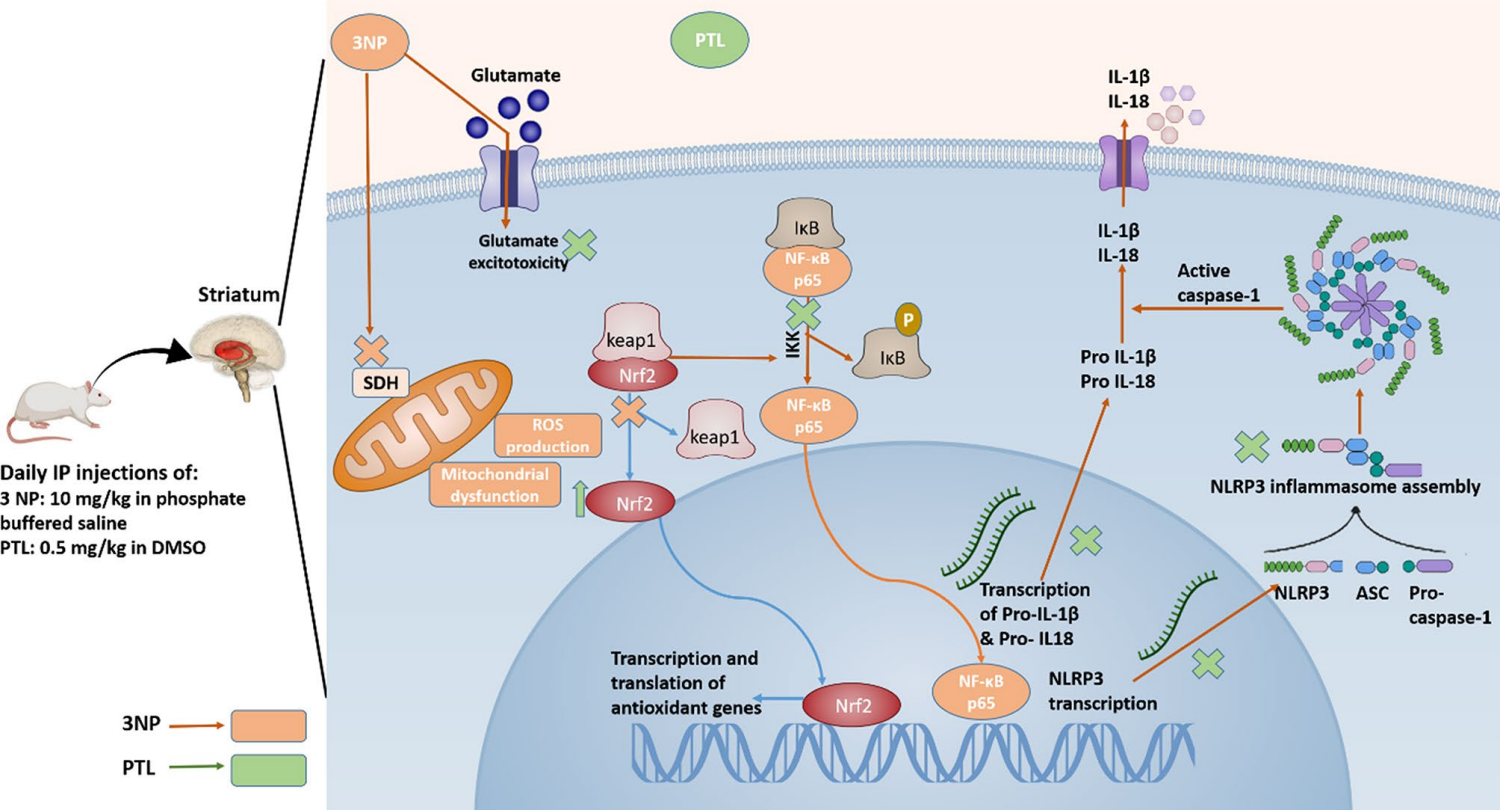
Schematic representation summarizing the neuroprotective effects of PTL in 3NP-induced HD-like perturbations in rats. 3NP: 3-nitropropionic acid, PTL: parthenolide, IP: intraperitoneal, DMSO: dimethyl sulfoxide, SDH: succinate dehydrogenase, ROS: reactive oxygen species, Nrf2: nuclear factor erythroid 2-related factor 2, Keap1: Kelch-like ECH-associated protein, NF-κB: nuclear factor kappa B, IκB: inhibitory-κB, IL-1β: interleukin-1β, IL-18: interleukin-18, NSE: neuron-specific enolase, GFAP: glial fibrillary acidic protein, C3: complement component 3, S100A10: S100 calcium-binding protein A10, CD45: cluster of differentiation 45, Iba1: ionized calcium binding adapter molecule-1

In conclusion, the present study is the first demonstration of the anti-Huntington effect of PTL by exerting a neuroprotective effect against 3NP-induced HD-like symptoms and its associated impairments in the striatum. The neuroprotective effect of PTL could be accredited to its antioxidant and anti-inflammatory properties via Nrf2 activation and inhibition of NF-κB expression along with targeting NLRP3 inflammasome activation and mitigating neuroinflammation. The NLRP3 inhibitor, PTL, not only restrained NLRP3 and NF-κB activation, but also enhanced neuronal survival and attenuated astrogliosis and microgliosis caused by 3NP, as summarized in Fig. 10. Our findings emphasize the significance of NLRP3 inflammasome inhibition as a potential therapeutic approach for HD and offer promising perspectives for future studies and clinical applications of PTL.

## Electronic supplementary material

Below is the link to the electronic supplementary material.

Supplementary Material 1

## Data Availability

All data that support the conclusions of the present study are available from the corresponding author upon reasonable request.
