# Supplementary material for: Parthenolide ameliorates 3-nitropropionic acid-induced Huntington’s disease-like aberrations via modulating NLRP3 inflammasome, reducing microglial activation and inducing astrocyte shifting
Source: Mol Med. 2024 Sep 26;30:158. doi: 10.1186/s10020-024-00917-5 (PMC11425901; doi:10.1186/s10020-024-00917-5)
Supplement: Supplementary file 1 — Supplementary Material 1 [file 10020_2024_917_MOESM1_ESM.docx]

**Supplementary Table 1. Correlation between astrocyte markers and the studied parameters**

|  | | **GFAP** | **C3** | **S100A10** |
| --- | --- | --- | --- | --- |
| **NLRP3** | ***r*** | 0.743478261 | 0.77960928 | 0.041514042 |
|  | ***p*** | **3.13922E-05** | **1.64386E-06** | 0.837107812 |
| **ASC** | ***r*** | 0.503478261 | 0.77025641 | -0.365689866 |
|  | ***p*** | **0.012137276** | **4.18027E-06** | 0.060678194 |
| **Caspase 1** | ***r*** | 0.677391304 | 0.856532357 | -0.11001642 |
|  | ***p*** | **0.00027671** | **1.1979E-08** | 0.577317754 |
| **Nrf2** | ***r*** | -0.37565217 | -0.55538462 | 0.414615385 |
|  | ***p*** | 0.070453617 | **0.003950972** | **0.039327414** |
| **Keap 1** | ***r*** | 0.55533813 | 0.801526755 | -0.205496193 |
|  | ***p*** | **0.004846419** | **5.04582E-07** | 0.303813433 |
| **NF-κB** | ***r*** | 0.588695652 | 0.762307692 | -0.508461538 |
|  | ***p*** | **0.002475762** | **9.47448E-06** | **0.009450281** |
| **IL-IB** | ***r*** | 0.594042197 | 0.43969995 | -0.199269094 |
|  | ***p*** | **0.002208183** | **0.027852169** | 0.339597719 |
| **IL-18** | ***r*** | 0.759130435 | 0.581538462 | -0.12026862 |
|  | ***p*** | **1.70168E-05** | **0.002295619** | 0.550147871 |

*NLRP3; nucleotide-binding domain leucine-rich repeat (NLR) and pyrin domain containing receptor, ASC; apoptosis-associated speck-like protein containing a caspase recruitment domain, Caspase 1, NF-κB; Nuclear factor kappa B, Nrf2; Nuclear factor erythroid 2-related factor 2, Keap1; Kelch-like ECH-associated protein 1, IL-1β; interleukin 1 beta, IL-18; interleukin 18, GFAP; glial fibrillary acidic protein, C3; complement component 3, S100A10; S100 calcium-binding protein A10.*

**Supplementary Table 2. Correlation between microglial markers and the studied parameters**

|  | | **CD45** | **Iba1** |
| --- | --- | --- | --- |
| **NLRP3** | ***r*** | 0.63882275 | 0.55555556 |
|  | ***p*** | **0.00077958** | **0.00321472** |
| **ASC** | ***r*** | 0.55875113 | 0.48173913 |
|  | ***p*** | **0.0045388** | **0.01714252** |
| **Caspase 1** | ***r*** | 0.66726348 | 0.6191453 |
|  | ***p*** | **0.00036839** | **0.00074516** |
| **Nrf2** | ***r*** | -0.25334135 | -0.33307692 |
|  | ***p*** | 0.23229535 | 0.10374412 |
| **Keap1** | ***r*** | 0.47747067 | 0.45825321 |
|  | ***p*** | **0.01829947** | **0.02123676** |
| **NF-κB** | ***r*** | 0.58412902 | 0.48173913 |
|  | ***p*** | **0.00272565** | **0.01714252** |
| **IL-IB** | ***r*** | 0.47243775 | 0.87296975 |
|  | ***p*** | **0.0197444** | **6.0047E-09** |
| **IL-18** | ***r*** | 0.56881477 | 0.85225885 |
|  | ***p*** | **0.00372567** | **1.6874E-08** |

*NLRP3; nucleotide-binding domain leucine-rich repeat (NLR) and pyrin domain containing receptor, ASC; apoptosis-associated speck-like protein containing a caspase recruitment domain, Caspase 1, NF-κB; Nuclear factor kappa B, Nrf2; Nuclear factor erythroid 2-related factor 2, Keap1; Kelch-like ECH-associated protein 1, IL-1β; interleukin 1 beta, IL-18; interleukin 18, CD45; cluster of differentiation 45, Iba1; ionized calcium-binding adapter molecule 1.*
